# Whole genome sequence data of *Lactiplantibacillus plantarum* IMI 507027

**DOI:** 10.1016/j.dib.2022.108025

**Published:** 2022-03-06

**Authors:** Ivana Nikodinoska, Jenny Makkonen, Daniel Blande, Colm Moran

**Affiliations:** a Alltech European Headquarters, Sarney, Summerhill Road, Dunboyne, Co. Meath, Ireland; b Biosafe-Biological Safety Solutions Ltd, Microkatu 1M, Kuopio, Finland; c Regulatory Affairs Department, Alltech SARL, Vire, France

**Keywords:** Lactic acid bacteria, Microbial genomics, Antimicrobial resistance, *Lactiplantibacillus plantarum*, Microbial bioinformatics

## Abstract

Here we report the draft genome sequence of the *Lactiplantibacillus plantarum* IMI 507027 strain. The genome consists of 37 contigs with a total size of 3,235,614 bp and a GC% of 44.51. After sequence trimming, 31 contigs were annotated, revealing 3,126 genes, of which 3,030 were coding sequences. The Average Nucleotide Identity (ANI) gave a value of 99.9926% between IMI 507027 and *L. plantarum* JDM1, identifying the strain as *L. plantarum*. No genes of concern for safety-related traits such as antimicrobial resistance or virulence factors were found. The annotated genome and raw sequence reads were deposited at NCBI under Bioproject with the accession number PRJNA791753.

## Specifications Table

**Table:** 

Subject	Microbiology
Specific subject area	Microbial genomics
Type of data	Raw reads of sequenced genome, assembled and annotated draft genome of *L. plantarum* strain IMI 507027
How the data were acquired	Illumina NovaSeq 6000, Unicycler v 0.4.8, Prokka v 1.14.5, NCBI Bacterial Antimicrobial Resistance Reference Gene Database v. 2021–06–01.1, ResFinder (downloaded on 20.04.2021), VFDB (Accessed on 14.07.2021), PlasmidFinder (2.1)
Data format	Raw
Description of data collection	Pure culture of *Lactiplantibacillus plantarum* IMI 507027 was used to isolate genomic DNA according to the Qiagen Genomic DNA Handbook (Qiagen) and Genomic-Tip 100/G (Qiagen) procedure. The genomic DNA was sequenced using NovaSeq 6000 Platform (Illumina). The raw reads were used for genome assembly, and the annotation, search of antimicrobial resistance genes, virulence factors, and plasmids were based on the assembled genome.
Data source location	Institution: Alltech Inc. City/Town/Region: Nicholasville, Country: Kentucky
Data accessibility	The data is hosted on a public repository. Bioproject: PRJNA791753 NCBI GenBank Accession Number: JAJTVG000000000 NCBI SRA Accession Number: SRR18032685 Direct URL to data: https://www.ncbi.nlm.nih.gov/sra/SRR18032685 Zenodo DOI Number for the annotated genome (gbk and gff files): 10.5281/zenodo.6123911.

## Value of the Data

• The lactic acid bacteria member *Lactiplantibacillus plantarum* is widely used to improve human and animal health. The present whole genome sequencing data describe the identity and safety-related features of a valuable agri-food isolate, namely *Lactiplantibacillus plantarum* IMI 507027.
• The reported data for the *L. plantarum* IMI 507027 isolate represents a great contribution for the fundamental as well as applied microbial research purposes.
• The sequencing data and the described microbial bioinformatics workflow can be used in lactic acid bacteria studies, e.g., comparative genomics, a search of antibiotic resistance genes, virulence genes, and plasmids in related microbial species.

## 1 Data Description

Here we report the whole genome sequencing data of *Lactiplantibacillus plantarum* IMI 507027, together with its safety-related features such as antimicrobial resistance and presence of virulence factors.

The whole genome sequence consisted of 37 contigs with a total size of 3235,614 bp, a GC% of 44.51, and an N50 contig length of 366,540 bp. The average sequencing coverage was 518x. During the annotation step, contigs below 200 bp in length were removed, obtaining an annotated assembly of 31 contigs (3,234,779 bp). The annotation produced 3126 genes, of which 3030 were CDS (coding sequences), 38 miscellaneous RNAs (non-categorised non-coding RNA), 2 rRNAs (ribosomal RNAs), 1 tmRNA (transfer-messenger RNA) and 55 tRNAs (transfer RNA). The NCBI Genome database contains 613 *L. plantarum* genome assemblies [search date: 16.7.2021]. The median total length is 3,253,870 bp, with a median protein (CDS) count of 2926 and median GC% of 44.5. Thus, the sequencing of IMI 507027 produced a complete genome (99.4%) comparable in size to the median genomic parameters for this organism. The 16S rRNA analysis, performed using the RDP Sequence Match against type strains, gave the highest similarity score to *L. plantarum* and other lactic acid bacteria. The alignment-free genome distance estimation analysis with Mash using MinHash evidenced *L. plantarum* JDM1 (GenBank accession number: CP001617) as the closest genome. The alignment-based calculation of average nucleotide identity (ANI) gave a value of 99.9926% between IMI 507027 and *L. plantarum* JDM1. The strain was unequivocally identified as *L. plantarum*.

Searches for antimicrobial resistance genes were made against different bacterial antimicrobial gene databases. According to European Food Safety Authority (EFSA) sequences with above 80% identity and 70% coverage should be considered for further analysis [1]. No antimicrobial resistance genes exceeding these threshold values were found. Similarly, no genes encoding potential virulence or pathogenicity factors were identified. One contig (contig_22) was identified as a potential plasmid.

## 2 Experimental Design, Materials and Methods

### 2.1 Extraction of DNA and whole genome sequencing

For the DNA extraction, 10 mL MRS Broth cultures were incubated aerobically at +30 °C for 16–17 h. Genomic DNA was extracted according to the sample preparation and lysis protocol described for gram-negative and some gram-positive bacterial samples in the Qiagen Genomic DNA Handbook (Qiagen) and purified according to the Genomic-Tip 100/G (Qiagen) procedure.

A standard genomic Illumina 150 bp paired-end library was produced from the chromosomal DNA and sequenced using Illumina NovaSeq 6000 sequencing technology at Eurofins genomics (Constance, Germany). Sequencing produced 6,225,531 raw reads and 1880,110,362 sequenced bases. The reads were trimmed using Trimmomatic v.0.38.1 [5] obtaining 5,795,718 paired reads and 1675,142,139 bases after trimming. The trimmed reads were assembled using Unicycler v 0.4.8 [6] with default settings. Gene predictions and functional annotations were performed using Prokka v. 1.14.5 (rapid prokaryotic genome annotation) [7].

### 2.2 Taxonomic identification of the strain

The following bioinformatics tools were used: SeqMatch v3 in RDP release 11.6 (Ribosome Database Project) [8] for 16S rRNA analysis; Mash using MinHash v. 0.1.1 [9] for alignment-free genome distance estimation, and OrthoANI v. 1.40 [10] for calculating average nucleotide identity.

### 2.3 Search for antimicrobial resistance genes and virulence factors

The IMI 507027 genome was screened against two antimicrobial resistance gene databases; the NCBI Bacterial Antimicrobial Resistance Reference Gene database (NCBI PRJNA313047; database version 2021-06-01.1) and the ResFinder database (downloaded on 20.04.2021) [11]. Searches against the NCBI database were performed using AMRFinderPlus v3.10.5 [12], ABRicate v 1.0.1 (https://github.com/tseemann/abricate; Seemann, 2014) and DIAMOND (Galaxy Version 0.9.29.0) [13]. AMRFinderPlus was run in combined mode performing searches on both the genome sequence and predicted protein sequences. ABRicate searches were performed on the nucleotide version of the same database for both the genome sequence and predicted gene sequences. The searches with DIAMOND were performed on the predicted protein sequences. The database searches were filtered at a minimum sequence identity of 80% and minimum coverage of 70%. Searches against the ResFinder database were performed using ABRicate v 1.0.1 and BLASTn (Galaxy Version 0.3.3). ABRicate searches were made on the genome sequence and predicted gene sequences. Search parameters included a minimum identity of 80% and minimum coverage of 70%. Since ResFinder is a nucleotide database, BLASTn searches were performed using the predicted gene sequences as a query.

Searches were performed against the virulence factor database (VFDB) [2,3] full dataset (Accessed on 14.07.2021).

### 2.4 Search for plasmids

Plasmids were searched from the genome data by screening the contigs against the PlasmidFinder database [4]. Assembly files were examined for circular contigs, and BLAST searches were conducted to identify if contigs were likely to be plasmids.

## CRediT authorship contribution statement

**Ivana Nikodinoska:** Data curation, Writing – original draft, Project administration. **Jenny Makkonen:** Methodology, Software, Writing – review & editing. **Daniel Blande:** Software, Formal analysis, Writing – review & editing. **Colm Moran:** Supervision, Project administration, Writing – review & editing.

## Declaration of Competing Interest

The authors declare that they have no known competing financial interests or personal relationships that could have appeared to influence the work reported in this paper.
